# Method of Measuring the Mismatch of Parasitic Capacitance in MEMS Accelerometer Based on Regulating Electrostatic Stiffness

**DOI:** 10.3390/mi9030128

**Published:** 2018-03-15

**Authors:** Xianshan Dong, Shaohua Yang, Junhua Zhu, Yunfei En, Qinwen Huang

**Affiliations:** 1 Science and Technology on Reliability Physics and Application of Electronic Component Laboratory, No.5 Electronics Research Institute of the Ministry of Industry and Information Technology, Guangzhou 510610, China; dongxs@pku.edu.cn (X.D.); yangsh@ceprei.com (S.Y.); zhujunhua@ceprei.com (J.Z.); enyf@ceprei.com (Y.E.); 2 College of Physics and Optoelectronic Engineering, Guangdong University of Technology, Guangzhou 510006, China

**Keywords:** MEMS accelerometer, mismatch of parasitic capacitance, electrostatic stiffness

## Abstract

For the MEMS capacitive accelerometer, parasitic capacitance is a serious problem. Its mismatch will deteriorate the performance of accelerometer. Obtaining the mismatch of the parasitic capacitance precisely is helpful for improving the performance of bias and scale. Currently, the method of measuring the mismatch is limited in the direct measuring using the instrument. This traditional method has low accuracy for it would lead in extra parasitic capacitive and have other problems. This paper presents a novel method based on the mechanism of a closed-loop accelerometer. The strongly linear relationship between the output of electric force and the square of pre-load voltage is obtained through theoretical derivation and validated by experiment. Based on this relationship, the mismatch of parasitic capacitance can be obtained precisely through regulating electrostatic stiffness without other equipment. The results can be applied in the design of decreasing the mismatch and electrical adjusting for eliminating the influence of the mismatch.

## 1. Introduction

An accelerometer is a key device in inertial navigation and control systems for measuring the acceleration information of a carrier. With the progress of MEMS technology, the MEMS accelerometer has been rapidly developed and is widely used in military, industry, medicine, and consumer electronics fields for its small volume, light weight, small power consumption, and low cost. Among MEMS accelerometers, the closed-loop capacitive accelerometer based on electrostatic force balance is an important form for its relatively good performance [1,2].

The MEMS capacitive accelerometer measures the acceleration through electrically detecting the changed differential capacitance of sensor caused by the movement of proof-mass under acceleration. As is known to all, parasitic capacitance is a serious problem in MEMS capacitive accelerometers [3,4,5]. Its mismatch between electrodes including in the sensor, package, and circuit would produce an offset and deteriorate the performance of bias and scale. The mismatch of effective capacitance due to process variation during sensor fabrication can be eliminated by the closed-loop system, but the mismatch of parasitic capacitance remains. Some research has been carried out for eliminating the influence of the parasitic capacitance [6,7,8], but these methods are either unsolved completely or lead to extra questions. Reducing the mismatch of parasitic capacitance is more direct and effective, and another solution is compensating the mismatch through electrical adjusting or adding an extra capacitor which is widely used [9,10]. Either reducing or compensating the mismatch of parasitic capacitance should be measured accurately.

Currently, the method of measuring the parasitic capacitance is limited in the direct measuring using the instrument or the capacitive measuring circuit [11,12]. This method has low accuracy for it would lead to extra parasitic capacitance and the measuring result is the state of off-power, moreover, some equivalent parasitic capacitance cannot be obtained and it cannot be implemented in some occasions. This paper proposes a novel method of measuring the mismatch of parasitic capacitance in MEMS accelerometer based on the mechanism of a closed-loop system. Through regulating the electrostatic negative stiffness and obtaining the curve between the output of electric force and the square of pre-load voltage, the mismatch can be obtained according to the coefficient of linear fitting. This method can be applied in the design for reducing the mismatch and electrical adjusting for eliminating the influence of mismatch, and the research for the characteristics of the mismatch influenced by the temperature and the self-calibrating technique of eliminating the mismatch can be further studied with this method.

## 2. Method of Measuring the Mismatch of Parasitic Capacitance

### 2.1. Influence of Parasitic Capacitance

**Figure 1:**
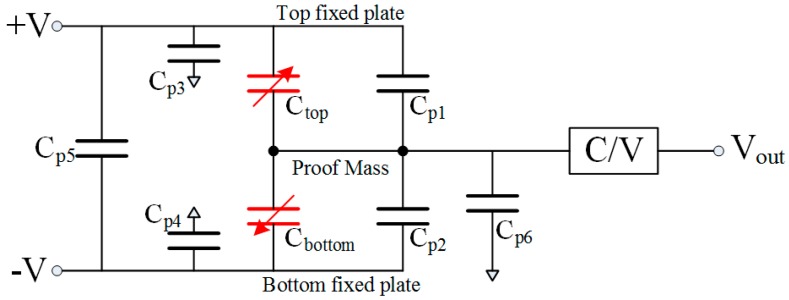
Schematic of capacitance in system of MEMS accelerometer.

Figure 1 shows the schematic of effective and parasitic capacitances in MEMS capacitive accelerometer interfaced with a C/V converting circuit. Obviously, there are several parasitic capacitances and the mismatch of parasitic capacitances ΔC_m1_ between C_p1_ and C_p2_—including in the sensor, package, and circuit—will confuse the differential effective capacitances ΔC between C_top_ and C_bottom_ that would produce an offset. The mismatch ΔC_m2_ between C_p3_ and C_p4_ will also have an influence on the output. Besides, the parasitic capacitances, C_p5_ and C_p6_, can affect the influence of ΔC_m1_ and ΔC_m2_ on the output.

Generally, the sensitivity of effective capacitance is about 100 fF/g or even smaller and the mismatch of parasitic capacitance can be up to 100 fF that will result in an offset of 1 g. This large offset would severely deteriorate the performance of the accelerometer. Therefore, it is necessary to study the mismatch and do some work for reducing the influence. Measuring the mismatch accurately is a basic step. Though there are many discrete parasitic capacitances, we only need to obtain the total equivalent mismatch.

### 2.2. Theory of Measuring the Mismatch

**Figure 2:**
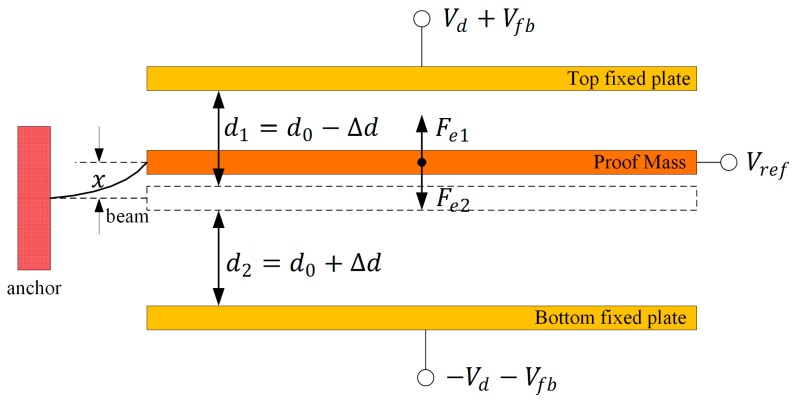
Sensor working diagram of electrostatic force balance.

In the closed-loop system of a MEMS capacitance accelerometer, there is electrostatic force between fixed plates and proof mass that balances the inertial force caused by acceleration [13], and the proof mass is not at the geometrical center for the mismatch of parasitic capacitance. Figure 2 shows a working diagram of the sensor.

Considering the process variation and parasitic capacitance, the electrostatic force $F_{e}$ of the proof mass is:

(1) $$F_{e}=F_{e1}-F_{e2}=\frac{\varepsilon_{r}\varepsilon_{0}A\times {\left(V_{d}+V_{fb}-V_{ref}\right)}^{2}}{2{\left(d_{0}-\Delta d-x\right)}^{2}}-\frac{\varepsilon_{r}\varepsilon_{0}A\times {\left(-V_{d}-V_{fb}-V_{ref}\right)}^{2}}{2{\left(d_{0}+\Delta d+x\right)}^{2}}$$

where $\varepsilon_{r}$ and $\varepsilon_{0}$ are the relative and absolute dielectric constant respectively, A is the overlapped area of capacitance, $V_{d}$ is the modulated voltage, $V_{fb}$ is the feedback voltage, $V_{ref}$ is the pre-load voltage, $d_{0}$ is the average gap between electrodes, Δd is the gap deviation due to process variation, and x is the bending value of the beam due to the mismatch of effective and parasitic capacitance.

In general, x and Δd are far smaller than $d_{0}$, and then, Equation (1) can be simplified to:

(2) $$F_{e}=\frac{2\varepsilon_{r}\varepsilon_{0}A\times V_{ref}V_{fb}}{d_{0}^{2}}-\frac{2\varepsilon_{r}\varepsilon_{0}A\times \left(V_{ref}^{2}+V_{fb}^{2}+V_{d}^{2}\right)}{d_{0}^{3}}\times \left(x+\Delta d\right)$$

where the bending value x consists of $x_{1}$ brought by the mismatch of effective capacitance and $x_{2}$ brought by the mismatch of parasitic capacitance, so $x=x_{1}+x_{2}=-\Delta d+x_{2}$. Substituting this equation to Equation (2), the electrostatic force $F_{e}$ can be expressed as:

(3) $$F_{e}=\frac{2\varepsilon_{r}\varepsilon_{0}A\times V_{ref}V_{fb}}{d_{0}^{2}}-\frac{2\varepsilon_{r}\varepsilon_{0}A\times \left(V_{ref}^{2}+V_{fb}^{2}+V_{d}^{2}\right)}{d_{0}^{3}}\times x_{2}$$

where $2\varepsilon_{r}\varepsilon_{0}A\times \left(V_{ref}^{2}+V_{fb}^{2}+V_{d}^{2}\right)/d_{0}^{3}=k_{e}$ is called electrostatic stiffness.

In the closed-loop system, there is the force balance for the proof mass:

(4) $$F_{e}+kx+ma+F_{s}=0$$

where k is the stiffness of the beam, m is the inertial mass of the proof mass, a is the external acceleration, and $F_{s}$ is the residual stress. Replacing Equation (3) into Equation (4), the formula of force balance can be expressed as:

(5) $$\frac{2\varepsilon_{r}\varepsilon_{0}A\times V_{ref}V_{fb}}{d_{0}^{2}}-\frac{2\varepsilon_{r}\varepsilon_{0}A\times \left(V_{ref}^{2}+V_{fb}^{2}\right)}{d_{0}^{3}}\times x_{2}=B_{0}$$

where $B_{0}=2\varepsilon_{r}\varepsilon_{0}A\times V_{d}^{2}\times x_{2}/d_{0}^{3}-kx-ma-F_{s}$. When the input acceleration is unchanged, the parameter $B_{0}$ can be considered as a fixed value. When the input acceleration and offset are small, $V_{fb}^{2}$ is far smaller than $V_{ref}^{2}$, so Equation (5) can be simplified to:

(6) $$\frac{2\varepsilon_{r}\varepsilon_{0}A\times V_{ref}V_{fb}}{d_{0}^{2}}=\frac{2\varepsilon_{r}\varepsilon_{0}A\times x_{2}}{d_{0}^{3}}\times V_{ref}^{2}+B_{0}$$

For the digital acquisition system, the left portion in Equation (6) can be transformed to $F_{e}^{\prime }=2\varepsilon_{r}\varepsilon_{0}A\times V_{ref}V_{fb}/d_{0}^{2}=U_{out}/K_{1}\times m\times g_{L}$ where $U_{out}$ is digital output which unit is LSB, $K_{1}$ is the scale of accelerometer which unit is LSB/g and $g_{L}$ is local gravity acceleration. Then, Equation (6) can be transformed to:

(7) $$\frac{U_{out}}{K_{1}}\times m\times g_{L}=\frac{2\varepsilon_{r}\varepsilon_{0}A\times x_{2}}{d_{0}^{3}}\times V_{ref}^{2}+B_{0}$$

Equation (7) can be transformed to:

(8) $$Y=B_{1}\times X+B_{0}$$

where $Y=U_{out}/K_{1}\times m\times g_{L}$ is dependent variable, $X=V_{ref}^{2}$ is independent variable, $B_{1}=2\varepsilon_{r}\varepsilon_{0}A\times x_{2}/d_{0}^{3}$ is linear coefficient and $B_{0}$ is intercept which is a fixed value.

Equation (8) shows that the relationship between output of electrostatic force $F_{e}^{\prime }=U_{out}/K_{1}\times m\times g_{L}$ and the square of pre-load voltage $V_{ref}^{2}$ is linear. Thus, we can make a curve with $F_{e}^{\prime }$ as *y*-axis and $V_{ref}^{2}$ as *x*-axis, and then, a linear fitting of the curve is made. Lastly, the mismatch of the parasitic capacitance can be obtained from the linear coefficient $B_{1}$ through the equation:

(9) $$\Delta C_{p}=\frac{\varepsilon_{r}\varepsilon_{0}A}{d_{0}-x_{2}}-\frac{\varepsilon_{r}\varepsilon_{0}A}{d_{0}+x_{2}}\approx \frac{2\varepsilon_{r}\varepsilon_{0}A\times x_{2}}{d_{0}^{3}}\times d_{0}=B_{1}\times d_{0},$$

where $d_{0}$ can be calculated through the obtained scale of the closed-loop system. Meanwhile, we can get the offset and the deviation from geometrical center due to the mismatch of parasitic capacitance.

## 3. Measurement Results and Discussion

Measuring tests have been done with closed-loop MEMS accelerometer to verify this novel method and two applications with this method are present. The measuring work were implemented on a printed circuit board (PCB) with discrete component, interfaced with a packaged sensor using ceramic shell and bond wire. The senor is fabricated with bulk silicon process and the structure is comb finger. The control system is achieved by analogue circuit and the analogue output is digitally acquired through Analog to Digital Convert (ADC) and Field Programmable Gate Array (FPGA) chip. The full-scale range of the accelerometer is 30 g, and the noise is 10μg/√Hz. In this system, the parasitic capacitances originate from the sensor, the ceramic shell, the bond wire and the PCB circuit. In our designed accelerometer, this mismatch commonly leads in an offset of several hundred mg that severely deteriorates the performance of accelerometer.

### 3.1. Measurement Results

#### 3.1.1. Verification Experiment and Results

In the verification experiment, the accelerometer is placed on the marble platform and the input acceleration is about 0 g which purpose is to make the external acceleration stable and the output very small. This step can improve the accuracy of the measurement. Because the pre-load voltage goes through voltage follower and resistance, and then reaches the node of proof-mass, so, the pre-load voltage does not directly connect to this C/V node. We draw out a line from the node of pre-load voltage that did not change the output. Then, the pre-loaded voltage of the accelerometer is changed, and the scale is tested through turning the accelerometer. The changed pre-loaded voltage, the digital output and the scale are record. Table 1 contains the measuring data with different pre-loaded voltage.

**Figure 3:**
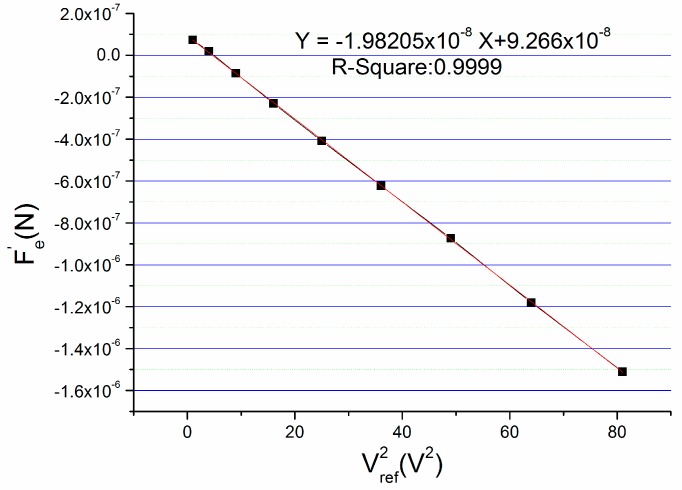
Relationship between $V_{ref}^{2}$ and $F_{e}^{\prime }$.

Using these recorded data, we make a figure by taking $V_{ref}^{2}$ as *x*-axis and $F_{e}^{\prime }$ as *y*-axis as shown in Figure 3, and a linear fitting of the curve is made.

The R^2^ of the linear fitting is 0.9999 which shows highly linear correlation between $V_{ref}^{2}$ and $F_{e}^{\prime }$. The strong linear relationship validates the theory of formula deduction. From the linear fitting formula, the linear coefficient can be obtained which is −1.98205 × 10^−8^. Through calculation according to this number, the bending value $x_{2}$ of the beam owing to the mismatch of parasitic capacitance which is also the deviation from the geometrical center is −13.48 nm. It should be noted that the bending value of the beam is a vector. That is to say it can be positive or negative. The bending direction of the beam depends on the sum of $x_{1}$ and $x_{2}$, and the minus sign of this $x_{2}$ indicates that the beam bends to the bottom plate, owing to the mismatch of parasitic capacitance. Correspondingly, the mismatch of parasitic capacitance is −69.372 fF and the offset caused by the mismatch is 219 mg.

#### 3.1.2. Applications and Results

The charge amplifier and diode ring are the common used C/V converting circuit. Because the charge amplifier is based on current measurement, the parasitic capacitance Cp3 and Cp4 in figure1 has little influence on the output of charge amplifier. However, in our design the diode ring detecting circuit is adopted for its simple structure. In diode ring detecting circuit, the principle of C/V converting is based on charge-discharge of capacitance. The capacitance Cp3 and Cp4 would affect the charge–discharge process of demodulating capacitance, so, it has an effect on the output. We carried out an experimental test to study the influence on output of capacitance to ground (GND) previously. A 1 pF difference between C_p3_ and C_p4_ was made in MEMS accelerometer using diode ring detecting circuit and a change of 0.5 g on output was observed, so it is necessary to study the influence of the parasitic capacitance between the fixed plate and GND. It should be noted that the effect of this equivalent mismatch on output is not equal to the effective differential capacitance, so its equivalent mismatch cannot be measured using the direct measuring method. The experiment for measuring the equivalent mismatch of the parasitic capacitance between the fixed plate and GND is carried out.

**Figure 4:**
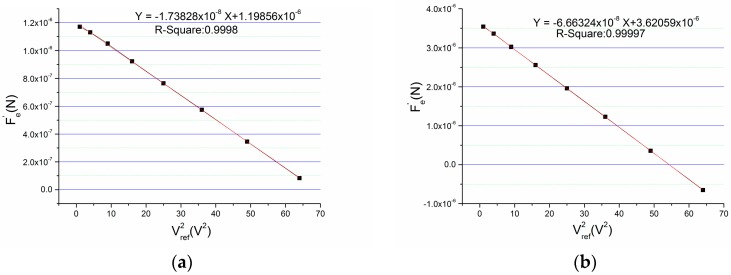
Measuring the equivalent mismatch between fixed plate and GND: (**a**) the initial state; (**b**) state of adding a capacitance of 1 pF.

A chip capacitor of 1 pF is intentionally added between the top fixed plate and GND. Because this operation changes the bias of accelerometer which is equivalent to changing the equivalent mismatch. Then, the total equivalent mismatch is measured using this novel method before and after adding this capacitor. Figure 4 is the testing results which show the influence of this mismatch.

The linear coefficient after adding the chip capacitor is much bigger than the one of initial state that indicates the parasitic capacitance between the fixed plate and GND can seriously affect the output. Through calculating, the initial mismatch of the accelerometer is −60.840 fF and the mismatch after adding the 1 pF capacitance is −233.213 fF. So, the equivalent mismatch of the 1 pF capacitance between the fixed plate and GND is 172.373 fF.

Another application using this method is improving the design of circuit to reduce the mismatch of parasitic capacitive. Table 2 shows the mismatch of parasitic capacitive for different sensors on same circuit board. For these six sensors, the average bending value $x_{2}$ is −11.0 nm and the average mismatch is −56.44 fF, which causes an offset of 179 mg. It can be seen that the values of the mismatch are near that indicates the mismatch is mainly from the circuit board for the mismatch of different sensors would have large discreteness.

**Figure 5:**
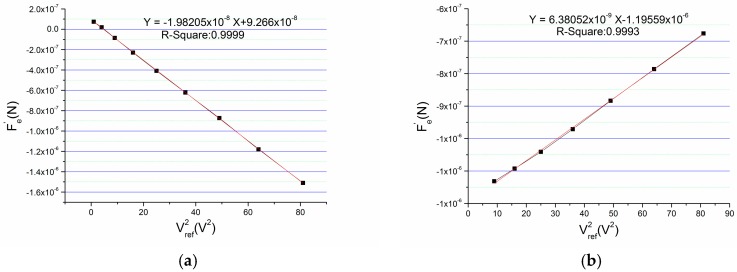
Mismatch of different circuit design: (**a**) result of before-optimization circuit; (**b**) result of after-optimization circuit.

The design of the circuit should be improved to reduce the mismatch of parasitic capacitance on the circuit board. An improved circuit was fabricated and the mismatch is measured with the same sensor welded on different circuit boards. Figure 5 is the contrast of mismatch on different circuit boards. The mismatch of parasitic capacitance is −69.372 fF on the before-optimization circuit board, and it is +22.332 fF on the after-optimization circuit board. It can be seen that through optimizing the circuit design, the mismatch of parasitic capacitance is reduced by 69% and the sign of the mismatch is changed.

### 3.2. Discussion

The linear relationship between output of electrostatic force and the square of pre-load voltage is validated by the experiment. In an ideal system with no mismatch, the force $F_{e}^{\prime }$ is a fixed value for the feedback and pre-load voltage are changed at inverse proportions. However, due to the existence of the mismatch of parasitic capacitance in real system, the force $F_{e}^{\prime }$ will be changed in proportion to $x_{2}$ following the changed force $k_{e}x_{2}$ when regulating the electrostatic stiffness through changing the pre-load voltage. The novel method exploits this characteristic to obtain the mismatch of parasitic capacitance.

It should be pointed out that the curve deviates from the straight line when the pre-load voltage is small, especially when the mismatch is small. This is because the force $k_{e}x_{2}$ has little change with a small pre-load voltage or a small mismatch that makes the linear relationship disturbed by the feedback voltage. Nevertheless, the mismatch of parasitic capacitance can be obtained precisely through regulating electrostatic stiffness with relatively high pre-loaded voltage. 

The measured results show the mismatch of capacitance parasitic is fF level. The mismatch is so small that requires testing equipment of very high precision. Different from the traditional methods, in this novel method a line is just drawn out from the pre-loaded node which does not interfere with any electrical node of the C/V frond-end circuit, so it does not introduce additional parasitic capacitance. Moreover, the measured result is the equivalent mismatch of all parasitic capacitance when the accelerometer is in an operating state. Therefore, the mismatch result is that we want.

## 4. Conclusions

This paper describes a novel method for measuring the mismatch of parasitic capacitance in MEMS capacitive accelerometer. The strong linear relationship between output of electrostatic force and the square of pre-load voltage is validated by the theory and experiment. The total equivalent mismatch of parasitic capacitance can be obtained precisely and conveniently through regulating electrostatic stiffness with changing the pre-loaded voltage. The results can be used in the design and electrical adjusting for decreasing the influence of the mismatch that is helpful for improving the performance of accelerometer, and the temperature characteristics of the mismatch and the self-calibrating technique of eliminating the mismatch can be further studied with this method.

## Figures and Tables

**Table 1:** The measuring data with different $V_{ref}$.

$V_{ref}\;(V)$	$U_{out}\;(\mathrm{LSB})$	$K_{1}\;(\mathrm{LSB}/g)$	$V_{ref}^{2}\;(V^{2})$	$F_{e}^{\prime }\;(N)$
1.00	5058	137,837	1.00	7.33 × 10^−8^
2.00	1526	68,051	4.00	1.96 × 10^−8^
3.00	−49	45,092	9.00	−8.48 × 10^−8^
4.00	−1079	33,768	16.00	−2.29 × 10^−7^
5.00	−1946	26,993	25.00	−4.09 × 10^−7^
6.00	−2651	22,491	36.00	−6.22 × 10^−7^
7.00	−3329	19,253	49.00	−8.73 × 10^−7^
8.00	−4043	16,811	64.00	−1.18 × 10^−6^
9.00	−4682	14,942	81.00	−1.51 × 10^−6^

**Table 2:** Mismatch of different sensors on same board.

Sensor	X_2_ (m)	Mismatch/fF
1	−1.12 × 10^−8^	−57.64
2	−0.99 × 10^−8^	−50.95
3	−0.98 × 10^−8^	−50.43
4	−1.14 × 10^−8^	−58.67
5	−1.17 × 10^−8^	−60.21
6	−1.18 × 10^−8^	−60.73
average	−1.10 × 10^−8^	−56.44
